# Modelling Innovation competence profiles: the empowering roles of self-monitoring and resilience

**DOI:** 10.1186/s40359-023-01340-x

**Published:** 2023-09-27

**Authors:** Kolawole Shola Ojo, Natalia V. Volkova

**Affiliations:** Department of Management, HSE University, Kantemirovskaya Str., 3, Bd., St. Petersburg, 1194100 Russia

**Keywords:** Innovation competence, Individual resilience, Self-monitoring, Latent Profile Analysis, Students

## Abstract

**Background:**

Innovation competence has been found to constitute distinct innovative abilities that must be analyzed together to get a more comprehensive picture of their effectiveness in various targeted groups. Drawn from the componential theory of creativity, such personal traits as individual resilience and self-monitoring play a critical role in developing innovation competence across students. This research aims to investigate the innovation competence profiles of students from various educational levels and study the role of individual resilience and self-monitoring in predicting the memberships of these profiles.

**Methods:**

A cross-sectional survey was conducted among university and college students, studying in a metropolitan area of the North-West region. The sampling scheme was stratified by the level of education and age. The questionnaire included items on the participants’ demographics, including gender, age, and level of education, measures of innovative abilities, individual resilience, and self-monitoring. This study sought to create innovation competence profiles in the student population using latent profile analysis. Multinomial logistic regression was employed to identify the impact of individual resilience and self-monitoring on innovation competence profile membership.

**Results:**

A sample of 638 university and college students was analyzed. The latent profile analysis classified students into three different innovation competence profiles - strong, moderate, and weak - with college and female students being identified as the typical members of the weak profile. Individual resilience increases the odds of membership into the strong profile than to moderate and weak profiles. High self-monitors have higher chances of being profiled into the strong profile than the weak and moderate profiles compared to the low self-monitors.

**Conclusions:**

Training investment aimed at boosting the innovative abilities of employees should consider the innovation competence profile of the beneficiaries to inform decisions about the appropriate level of intervention required. Likewise, educators could enrich their courses devoted to improving the innovative abilities of students with content that aims to improve their level of resilience accompanied by social support. Theoretical and practical implications are also discussed.

## Background

Owing to its developmental potential for national economies and industries, innovation has received a lot of recent attention. The emphasis has been on innovation as the engine of economic expansion and as a source of competitive advantage for organizations and individuals [1–3].

Although innovation and inventive projects are typically considered to be relevant at the organizational level, the concept’s foundation lies with the people in charge of them [4], [5]. At the individual level, innovation-related activities have become a significant part of many job functions in service-oriented (medical and health-related services, consulting and the like) or engineering occupations [2], [6]. Further, the success of any projects depends on the availability of individuals with high *innovation competence* - the ability to invent, introduce, adapt, and/or implement advantageous novelty at any organizational level [7]. Consequently, young professionals are in high demand for employers that have the necessary requirements for their skills to be put into practice for the development of corporate innovative initiatives [8]. It is therefore essential that universities offer courses that enhance the development of students’ innovative abilities required for working life [6].

In fact, higher education institutions and colleges play a crucial role in bridging the skills gap in light of the demands for innovative workers placed by the 21st-century labor market, Industry 4.0, and the Sustainable Development Agenda [8]. Having a particular set of abilities, such individuals demonstrate innovative work behavior defined as the creation and delivery of new products and/or services or the implementation of the change process itself to benefit a work role, group, or organization [9]. However, systematic literature reviews on innovation in the workplace indicated a dearth of research from the individual perspective, specifically with emphasis on innovation competence that mirrors employee behaviors to pursue innovative strategies [4], [10]. Hence, in the literature, several gaps exist in this field that need to be addressed to gain a more coherent picture of individual innovation competence and its connection with personal characteristics enabling people in ambitious projects under adverse market conditions and/or dynamic environments.

The first gap is that most research on innovation competence does not consider the heterogeneity of individual profiles but rather focuses mostly on a variable-centered approach to explore this subject. Typically, these studies investigate the underlying relationships between innovative abilities and various associated personal and job-related predictors, such as role performance [3], leadership styles [11], [12], personality traits [5], self-monitoring [13], and overall satisfaction with life [14]. More so, current research continues to provide evidence for additional factors that influence innovation competence, such as student learning performance [15], cultural intelligence [16], or learning experiences [6]. However, these studies have not adequately addressed the profiles of innovation competence in students by adopting a person-centered approach. Much can be learned about the potential involvement of such student groups in the innovation process. These profiles can be part of the basis for university course development (e.g., design of educational strategies for project-based courses) or HR practices (e.g., training or development) to foster innovative activities in students. Against this backdrop, the first objective of this study is to identify their profiles of innovation competence.

The next significant gap is associated with the heterogeneous connections of students belonging to different innovation competence profiles and the personal psychological attributes preparing them for complex and adverse situations. In fact, innovation projects are never simple and frequently fail; therefore, team members’ resilience, or the capacity to bounce back and recover from setbacks [17], is essential for the success of innovative endeavors [1], [18]. Likewise, because individual invention activities present a variety of difficulties, persons engaged in them can develop a high degree of resilience [19], [20]. Further, innovation development is a social process [12] because these activities typically involve people working together to solve a problem [21]. Thus, the ability of individuals to manage their expressive behaviors in social settings - self-monitoring - might have implications for their innovativeness. However, previous studies have not adequately addressed the characteristics of resilience and self-monitoring in determining the composition of innovative abilities in students. Therefore, the second objective of this study is to relate the innovation competence profiles obtained to the level of individual resilience and self-monitoring.

Based on the componential theory of creativity [22], [23], this study investigates the relationship between individual resilience, self-monitoring, and innovation competence profiles among the population of students and uses latent profile analysis and multinomial logistic regression to address both gaps by answering the core questions as follows:

### RQ1

How many different innovation competence profiles exist among university and college students?

### RQ2

How do the levels of self-monitoring and individual resilience predict membership of the different innovation competence profiles in students?

Using person-centered analysis to identify innovation competence profiles, this study contributes to the literature on the topics of innovation and human resource management in two ways. First, students demonstrated the various degrees of innovation competence expressed in three profiles. Second, individual characteristics, such as self-monitoring and resilience strengthen the evidence for the development of individual innovation capacity for both university and college students. In this way, university professionals and HR practitioners can assess and leverage the innovation competence profiles to develop more precise educational programs and employee capacity development initiatives respectively, for facilitating innovation involvement in the targeted groups.

### The assessment of innovation competence

The innovation process encompasses various activities for inventing, developing and commercializing new products and services, openings of new markets, changes in production methods, suppliers, business or management models [2], [24]. It develops through phases of problem recognition, generation and implementation of new and useful ideas [10], [11]. As such, various types of resources, particularly human, are needed for the increase of innovations [3].

Approaching from an innovative work behavior perspective, several authors argue that innovation requires the development of distinct competencies, skills and capacities that make up innovation competence [4], [7], [25]. According to Marín-García et al. [24], competency is formed by a set of capacities which integrate a number of skills requiring procedural and conditional knowledge for innovation. Unsurprisingly, individual competencies that combine knowledge, skills, capacities, and motivation are critical to facilitate innovation projects [6], [8].

In the literature, there are several models for assessment of innovation competence, such as the Four C model of creativity for education [26], Innovation Competencies Development project (INCODA) with emphasis on higher education [25], or Framework for Innovation Competencies Development and Assessment (FINCODA). In this study, we have followed a FINCODA innovation competence model focused specifically on behavioral indicators of innovators in working life. Further, this classification was developed for both employees and students to gauge their innovative abilities and, therefore, can indicate a skills gap in transition between university and the workplace [4].

The FINCODA innovation competence model is classified into five dimensions. The first two of these dimensions – creativity and critical thinking – are associated with abilities for inventive work phases of innovation and the last three dimensions – networking, initiatives and teamwork – are essential abilities for implementation phases of innovation [7]. As such, this model captures individual abilities relevant to the main stages of the innovation process. More specifically, understanding the composition of innovation competence dimensions by adopting person-centered analysis can bring new insight into innovative work behavior as it results in sub-groups (profiles) in which individuals differ significantly from the other sub-groups. However, to the best of our knowledge, no research has yet investigated such innovation competence profiles in students.

### The relationship of resilience and innovation competence

Individual resilience, a psychological attribute, refers to the capacity to make positive adjustments in the midst of stressful and adverse events, manage impacts of stress, adapt to change and adversity, and maintain an optimistic disposition [27], [28]. Resilient individuals can find an order in the middle of chaos, are risk-oriented, and are willing to be flexible and adaptive [20], [29]. Resilience is not just the capacity to cope, but it also includes growth and positive adaptation following difficult life challenges. Above all, as a developmental process, it is believed to be acquirable as against a hard-wired personality trait [30–32].

A literature review showed promising evidence that individual resilience is associated with various dimensions of innovation competence. Carmeli et al. [18] reported a positive effect of team members’ resilience on the team’s creativity in solving problems, which, in turn, became the key proximal predictor of project performance. Further, resilience positively impacts creative performance, sustaining creativity rather than initiating it, that is why resilient employees prepare ahead of hardship and are more committed to change and display change-oriented behavior [33]. Fandiño et al. [20] found that resilience positively promotes organizational innovation, which is strengthened by social capital. Bricolage and improvisation – a dimension of resilience – enhance social interaction, which in turn improves the innovation process. For example, the study cited how the Filipino immigrants, after Typhoon Milenyo, were able to leverage their social network to enhance the innovation process needed to improve the quality of their lives. Likewise, Caniëls et al. [19] reported the indirect and positive role of resilience on innovative work behavior through positive emotions. In the mechanism path, resilience positively supports the process of closing the gap between the current state (when a problem needs to be solved) and a desired state (where the problem is actually solved). Further, it is explained that the achievement of a solution produces feelings of satisfaction, joy, enthusiasm, and alertness – positive moods–which in turn increase idea generation (creativity). More so, the study concludes that paying attention, good social interactions and networking are necessary conditions that support both the idea generation and implementation stages of the innovation process, and that positive moods create these conditions. Hence, resilience increases teamworking, networking and creativity in solving problems, which naturally requires critical thinking and initiative.

Evidently, resilience should be more outstanding in innovative individuals as they are generally more mindful [31], maintain a positive metal health, affecting their productivity and positive regard toward coworkers [29], tend to demonstrate a higher level of social capital that leads to an atmosphere of goodwill and openness among individuals [20], and experience positive emotions more frequently [19].

### The relationship of self-monitoring and innovation competence

Self-monitoring has been defined as an aggregate construct that represents a combination of skills and motivation to gauge the extent to which individuals can adapt their expressive behavior to suit a situation [34], [35]. Generally, self-monitoring characteristics can be displayed in two continua: high and low self-monitoring [36], [37]. High self-monitors (HSM) modify their behaviors to conform to situational demands in order to gain a positive image, the approval of others, and enhance their social status. Conversely, low self-monitors (LSM) do not justify self-imaging and, therefore, are less apt to adjust their behavior to achieve approval, but rather act as they think and feel [13] HSM might have larger social networks and be more flexible and accommodating in dealing with others [36]. Hence, they are more likely to become leaders and often enjoy higher performance ratings based on subjective opinions.

The innovative work behavior of HSM is driven by the expected outcomes of their image (e.g., positive, or negative). Sulistiawan et al. [13] reported that HSM employees are more likely to engage only in innovative activities which are expected to result in positive social image gains, otherwise they avoid risky innovative projects, with high chances of failure that might damage their status in social settings. Furthermore, in an experimental study of 500 students, Arne J Vet & Carsten K. Dreu, [38] investigated the moderating role of self-monitoring on the effect of thinking aloud on creativity and report that thinking aloud reduces the number of original ideas and this negative effect is more pronounced for the group with high sensitivity to what others think of them but with low ability to adapt to expectations. The study explained that when highly sensitive people think aloud during creative activity, like idea generation, they may attempt to adapt their expression to elicit positive evaluation from others who are observing them - this is called “spotlight effects”. The authors concluded that this increased evaluation apprehension would reduce HSM creative performance – that is, the originality of their ideas. Conversely, Zhou and Li [39] documented that a supervisor’s developmental feedback, boosting intrinsic motivation, positively predicts employee creativity, particularly for HSM. High self-monitors are more likely to develop creative behaviors than their low self-monitoring peers because the former can adjust their behavior and respond more actively to the developmental feedback information provided by their supervisors. Hence, self-monitoring is positively related to innovation competence in particular social-organizational work settings, where innovative work behavior is supported by leaders and coworkers.

### Conceptual framework

We draw on the componential theory of creativity [22], [23] to explain how individual resilience and self-monitoring affect the membership in innovation competence profiles.

First, the literature review showed the relationships between creativity, the phases of innovation and innovation competence [7], [11], [23], as doing so would provide a good background for our conceptual framework. While innovation is impossible without creativity (idea generation), the promotion and implementation of the new ideas do require some other additional abilities and individual behaviors to be successful [11]. Marin-Garcia et al. (2016) summarized these essential individual abilities as innovation competence and proposed the FINCONDA model to assess and develop them. Putting it together, creativity is the foundation of innovation, but novel ideas are not sufficient to complete the multi-phased innovation process. Beyond the problem recognition and idea generation phases, innovators need to possess a set of abilities (innovation competence) to create innovative value through the promotion and implementation phases.

Second, we assume that the dimensions of innovation competence, required for innovative work behavior [4], [21], must be analyzed together to get a more comprehensive picture of their effectiveness in various targeted groups. Using person-centered analysis, this study sought to create innovation competence profiles in the student population, based on the FINCODA innovation competence model [7].

Third, according to the componential theory of creativity, the work environment drives individuals’ behavior to innovate [23]. In fact, people react differently to various social and organizational factors instigating innovations, such as teamwork, the leadership style of employees’ supervisors, or a sense of challenges. Their reactions to a variety of external forces can be estimated through either self-monitoring or individual resilience. Self-monitoring, which is the tendency to deliberately behave in ways that meet the expectations of others and earn their admiration, could be a motive that drives individuals to engage in creative tasks [13]. Further, the social environment component of the theory also resonates with the concept of self-monitoring, which describes the extent to which individuals can monitor, adjust, and adapt their behavior to respond to the social milieu around them, while others use social situations to achieve favorable impressions [36], [39]. Likewise, resilience, the second independent variable in this study, is synonymous with self-discipline, perseverance, and high flexibility [19], [20], which are important features of the idea implementation stage of the innovation process [4]. The literature review above suggests that these psychological attributes are positively related to innovation competence. However, some contrasting predictions were depicted regarding the relationship between self-monitoring and the social-organizational work environment, but it is not the case for the student population who are relatively inexperienced workers. Thus, we hypothesize:

#### H1

A higher level of individual resilience will increase the likelihood of membership in profiles with higher innovative abilities.

#### H2

A higher level of self-monitoring will increase the likelihood of membership in profiles with higher innovative abilities.

**Fig. 1:**
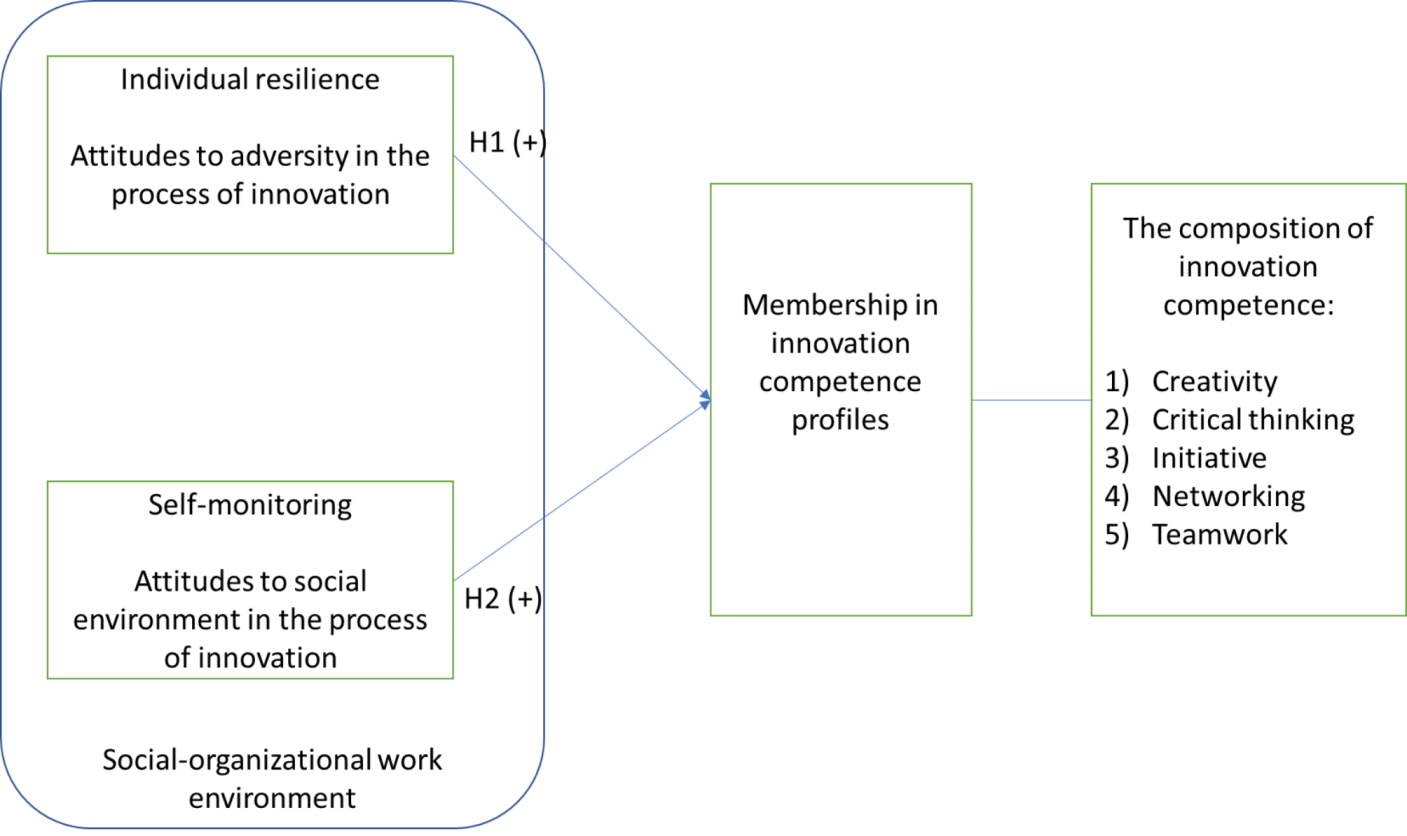
The relationships between resilience, self-monitoring, and innovation competence profiles

Moreover, the componential theory of creativity posits that individual personality is one of the factors that drives individual ability to innovate. We propose a framework that uses self-monitoring and individual resilience (both are personality traits) to predict the membership in innovation competence profiles. Figure 1 shows the visualization of this framework.

## Methods

### Participants and data collection

The data for this cross-sectional study were drawn from the universities and the colleges, located in a metropolitan area of the North-West region. The sampling scheme was stratified by the level of education and age. In the spring and fall semesters of 2022, the online self-report questionnaire was distributed across undergraduates and college students who were enrolled in the innovation management, organizational behavior, and/or entrepreneurial introductory courses. Recruitment channels included invitations via personal emails and social media platforms. Students consented to participate in the survey and completed the questionnaire anonymously and voluntarily. The respondents were advised that there were no right or wrong answers to the questions. No incentive was offered for participation. By the deadline established for data collection, 638 out of 1000 distributed questionnaires were completed, resulting in a response rate of 64%. The questionnaire consisted of demographic characteristics (gender, age, level of education), and assessments of self-monitoring, resilience, and innovative abilities. Responses to all assessments were anchored with a five-point Likert scale (1 = strongly disagree and 5 = strongly agree). All the scales were pretested, and the results confirmed their reliability for the Russian context.

### Demographic characteristics of students

The demographic characteristics were gender, age, and level of education. The level of education was divided into two groups as university (n = 267) and college (n = 371) students. The sample of each cohort was relatively homogeneous with respect to the average age, being 18 years old. The sample is 82.6% female and 58.2% students from colleges.

### Measures

#### The brief resilience scale

Resilience as the personal disposition to bounce back and flourish despite experiencing hardship was gauged by the unidimensional Brief Resilience Scale [17]. In this study, we used a 3-item questionnaire adopted by Charoensap-Kelly et al. [29] with the following statements: “I tend to bounce back quickly after hard times”, “it does not take me long to recover from stressful events”, “I tend to take a long time to get over setbacks in my life” (reverse-coded). These items were translated by three native speakers and cross-verified by other informants to keep translational integrity and linguistic similarity. The Cronbach’s alpha coefficient of this 3-item scale was 0.8.

#### The self-monitoring scale

The unidimensional self-monitoring-reduced scale was used to measure the psychometric properties of this psychological construct [37]. This questionnaire was utilized in Russian populations [40], [41]. The scale captured the ability to regulate the self-expressions of students for the sake of desired public appearance in social situations. Some of the statements in the scale are the following: “I guess I put on a show to impress or entertain people”, “In different situations and with different people, I often act like very differently”. Participants indicated their agreement or disagreement with 18 statements, resulting in the sum of answers in accordance with the keys. HSM individuals tended to answer in keyed directions (maximum score is 18). The Cronbach’s alpha reliability was 0.7 for this 18-item scale.

#### Innovative abilities

The innovative abilities were measured through the FINCODA project (2014–2017) funded by the European Union [8]. The 25-item questionnaire was adjusted to the local university students by Volkova and Plakhotnik [41] and consisted of five dimensions: creativity, critical thinking, teamwork, initiative, and networking. These subscales reflect the actions or behavior needed for different stages of innovation processes. The description and the number of the items for these FINCODA dimensions are the following:

1. *Creativity*: ability to transcend traditional ideas, patterns, rules, or relationships and generate or adapt alternatives independently of their possible practicality and future added value.
2. *Critical thinking*: ability to analyze and assess pros and cons and estimate the risks involved for a purpose.
3. *Initiative*: ability to make decisions to implement ideas that foster positive changes, as well as to mobilize creative people and those who must put the ideas into practice.
4. *Teamwork*: ability to work effectively in a group with collaboration.
5. *Networking*: ability to engage external/outside stakeholders in the team [7], [21].

### Statistical analysis

Statistical analysis was performed via R Studio. First, a multigroup confirmatory factor analysis was run to assess the appropriateness of the FINCODA questionnaire for college and university students. The goodness of fit of these models was analyzed using the following fit indices: RMSEA (the root-mean-square error of approximation), TLI (Tucker Lewis Index), CFI (the comparative fit index), and SRMR (Standardized Root Mean Square Residual). An RMSEA of 0.06 and SRMR of 0.08 or smaller, coupled with TLI and CFI values greater than 0.90, indicate an acceptable model fit [42].

Second, descriptive analyses were conducted to describe the frequency and percentage of the demographic characteristics, such as gender and the level of education. Pearson’s correlation analysis was applied to explore the bivariate association between the FINCODA dimensions, resilience, and self-monitoring.

Third, a latent profile analysis (LPA) was run to determine the number of profiles that exist in the data regarding innovative abilities and the nature of these profiles. LPA allows for identification of different sup-populations with different configural profiles of personal and/or environmental attributes within a population. Unlike other traditional non-latent clustering methods (like K-means clustering, hierarchical clustering), LPA treats profile membership as an unobserved category variable and membership of a profile is determined by latent variable value and the membership probability estimated from the LPA model [43]. Another strength of LPA is that it can model many types of variable categorical (ordinal, nominal), continuous variables [44]. To define the optimal number of profiles in the model, a series of latent profile models with increasing number of latent profiles was run by applying the tidyLPA package. The following statistical indicators were used to compare the k-profile model with the (k-1)- profile model iteratively: the Bayesian information criterion (BIC), the Akaike Information Criterion (AIC), the Consistent AIC (CAIC), log-likelihood, and p-values of the bootstrapped likelihood ratio test (BLRT). These indices were applied to select the final fitting model in which lower values of the BIC, AIC, and CAIC, coupled with higher log-likelihood values, determine an adequate solution [43]. In addition to these indicators, we evaluated the entropy value, theoretical coherence, and profile size to decide the final number of profiles. Higher entropy (up to the perfect value of 1) indicates better classification, demonstrating the accuracy of the model to assign individuals to the profiles. Any given profile should include a minimum of 1% of the sample or 25 cases in the profile [43]. All latent profiles must be meaningful and interpretable [45]. In this study, six models, specifying from one to six latent profiles, were estimated via robust full information maximum likelihood (MLR) estimation.

Finally, multinomial logistic regression was employed to identify the impact of resilience and self-monitoring on innovation competence profile membership.

## Results

### Multigroup confirmatory factor analysis

An initial multigroup confirmatory factor analysis (MGCFA) was performed via lavaan packages in R Studio to evaluate whether the five dimensions of the FINCODA were invariant to group membership and could be applied to both college and university students. Following the steps recommended by Davidov and others [46], we ensured, first, that the factor structures were equal across groups (configural invariance). The result showed that one item from the networking dimension had significantly unequal standardized regression weights across the two samples. This indicated that university and college students interpreted this item variously. A further MGCFA was conducted without this item. Next, the factor loadings of each item and the respective latent variable were set to be equal across the two groups (metric invariance). Then, the indicators of item intercepts were investigated (scalar invariance) in which, in accordance with Vandenberg and Lance’s suggestions [47], at least partial scalar invariance must be used for meaningful group comparisons. As evident in Table 1, no significance was found between the configural and metric models (χ2_Diff_ = 29.97, df = 19, p = 0.052) as well as the metric and partial scalar models (χ2_Diff_ = 21.7, df = 13, p = 0.06).

**Table 1:** Multigroup Confirmatory Factor Analysis

	chisq	df	RMSEA	TLI	CFI	SRMR
Model 1: configural invariance	873.25	474	0.051	0.925	0.935	0.047
Model 2: metric invariance	903.22	493	0.051	0.925	0.933	0.054
Delta Model 1 vs. Model 2	29.97	19			0.002	
Model 3: scalar invariance	1084.36	512	0.059	0.900	0.907	0.059
Delta Model 2 vs. Model 3	181.14***	19			0.026	
Model 4: partial scalar	924.92	506	0.051	0.926	0.932	0.054
Delta Model 2 vs. Model 4	21.7	13			0.001	

Notes: df - degrees of freedom; RMSEA -Root Mean Square Error of Approximation; SRMR - Standardized Root Mean Square Residual; TLI - Tucker Lewis Index; CFI - comparative fit index

*** - p < 0.001

Moreover, Cronbach’s alpha coefficients for the five dimensions of the FINCODA scale were acceptable, ranging from 0.7 to 0.8 (see Table 2). Finally, the 5-factor model for the FINCODA had acceptable fit statistics (χ2 = 605.911, df = 237, p < 0.000, TLI = 0.93, CFI = 0.94, RMSEA = 0.049, SRMR = 0.043), indicating that the current model supports the five latent factors of the FINCODA for both groups of students.

**Table 2:** *Means, standard deviations, Cronbach alpha coefficients and correlations with confidence intervals*

Variable		*M*	*SD*	α	1	2	3	4	5	6
1. Self-monitoring		9.63	3.17	0.7						
2. Resilience		3.12	1.06	0.8	0.09*					
					[0.02, 0.17]					
3. Creativity		3.75	0.69	0.8	0.16**	0.30**				
					[0.08, 0.23]	[0.23, 0.37]				
4. Critical thinking		3.71	0.82	0.8	0.16**	0.23**	0.74**			
					[0.08, 0.23]	[0.15, 0.30]	[0.70, 0.77]			
5. Initiative		3.58	0.77	0.8	0.17**	0.26**	0.72**	0.67**		
					[0.09, 0.24]	[0.19, 0.33]	[0.68, 0.76]	[0.63, 0.71]		
6. Teamwork		3.99	0.69	0.8	0.09*	0.17**	0.56**	0.58**	0.59**	
					[0.02, 0.17]	[0.10, 0.25]	[0.50, 0.61]	[0.52, 0.63]	[0.54, 0.64]	
7. Networking		3.93	0.73	0.7	0.15**	0.25**	0.57**	0.60**	0.61**	0.70**
					[0.07, 0.23]	[0.17, 0.32]	[0.51, 0.62]	[0.55, 0.65]	[0.56, 0.66]	[0.66, 0.74]

*Note.* Values in square brackets indicate the 95% confidence interval for each correlation. * indicates *p* < 0.05. ** indicates *p* < 0.01. α - Cronbach’s alpha

### Descriptive statistics and correlation analysis

Descriptive statistics, correlation, and Cronbach’s alpha coefficients of continuous variables are presented in Table 2. The self-monitoring had positive weak correlations with all five dimensions of innovative abilities (r = 0.16, 0.16, 0.17, 0.15 for creativity, critical thinking, initiative, and networking, respectively, p < 0.01, and r = 0.09, p < 0.05 for teamwork). The resilience demonstrated a bit higher positive correlations with innovative abilities in comparison with self-monitoring (r = 0.3, 0.23, 0.26, 0.17, 0.25 for creativity, critical thinking, initiative, teamwork, and networking, respectively, p < 0.01).

### Latent profile analysis

Latent profiles were examined using the mean scores of students’ five innovative abilities, which were standardized with a mean of zero and a standard deviation of one. In this analysis, the indicator variables were fixed to have zero covariances within and across profiles. The variances of the indicators are allowed to vary within profiles but are restricted to be equal between profiles. The fit statistics for the six LPA models are shown in Table 3, including the minimum number of cases in the profile. First, three models with four, five, and six profiles were rejected due to the occurrence of profiles with less than 25 cases of the sample. These findings suggested using a three-profile solution as the best model of these data, which presents an entropy value of 84.6%. This indicates that the three profiles are capable of accurately classifying all the samples in 84%. Finally, interpretability of the three-profile solution, with weak, moderate, and strong levels of innovative abilities, is more meaningful for practitioners.

**Table 3:** Data fit for LPA models

Profiles	LogLik	AIC	BIC	CAIC	Entropy	p-value of BLRT	Minimum number of cases
1	-4523.91	9067.82	9112.41	9122.41	1.00		
2	-3997.67	8027.35	8098.68	8114.68	0.81	0.01	299
3	-3765.03	7574.07	7672.15	7694.15	0.85	0.01	94
4	-3645.18	7346.35	7471.18	7499.18	0.86	0.01	18
5	-3605.86	7279.72	7431.30	7465.30	0.82	0.01	12
6	-3583.33	7246.66	7424.99	7464.99	0.76	0.01	12

**Fig. 2:**
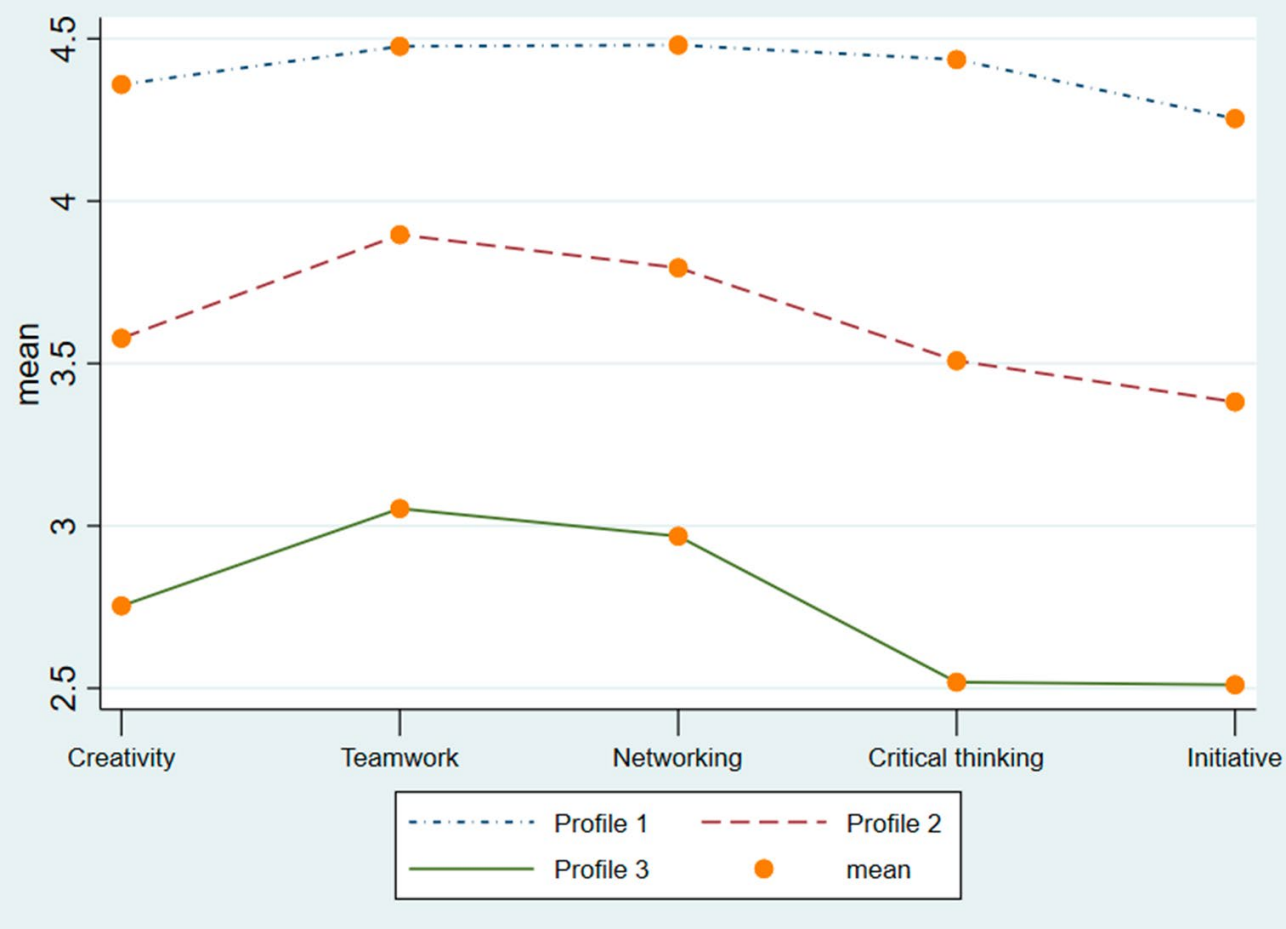
Profile plot Note. Profile 1: Strong innovative abilities; Profile 2: Moderate innovative abilities; Profile 3: Weak innovative abilities

We labeled the obtained three profiles as weak, moderate, and strong innovative abilities of students (see Table 4; Fig. 2). The distribution of the sample is the reference for the interpretation concerning what means weak, moderate, and strong [48].

*Profile 1*, or strong innovative abilities (n = 241; 37.8%), consists of respondents with the highest level of the five FINCODA dimensions, self-monitoring, and resilience in this sample, regardless of their education level and gender. The number of college and university students is about equal in this sub-group. *Profile 2*, or moderate innovative abilities (n = 303; 47.5%), includes almost half of all students in this sample, indicating the relatively common pattern across these participants. *Profile 3*, or weak innovative abilities (n = 94; 14.7%), corresponds to students with a comparatively low level of the five FINCODA dimensions in this sample. This sub-group accounts for 73.4% of college students, indicating the need for development of innovation competence, especially regarding initiative (M = 2.4, SD = 0.63) and critical thinking (M = 2.5, SD = 0.68).

**Table 4:** Innovation competence profiles

Variables	Strong innovative abilities (n = 241)	Moderate innovative abilities (n = 303)	Weak innovative abilities (n = 94)	Total (N = 638)	p value
Gender					0.020
Female	188 (78.0%)	254 (83.8%)	85 (90.4%)	527 (82.6%)	
Male	53 (22.0%)	49 (16.2%)	9 (9.6%)	111 (17.4%)	
Education					< 0.001
College	122 (50.6%)	180 (59.4%)	69 (73.4%)	371 (58.2%)	
University	119 (49.4%)	123 (40.6%)	25 (26.6%)	267 (41.8%)	
Self-monitoring					< 0.001
Mean (SD)	10.353 (3.100)	9.251 (3.098)	9.032 (3.258)	9.635 (3.169)	
Range	3.000–18.0	2.000–16.0	2.000–17.0	2.000–18.0	
Resilience					< 0.001
Mean (SD)	3.490 (0.992)	2.944 (1.005)	2.720 (1.094)	3.117 (1.056)	
Range	1.000–5.000	1.000–5.000	1.000–5.000	1.000–5.000	
Creativity					< 0.001
Mean (SD)	4.359 (0.396)	3.578 (0.404)	2.754 (0.534)	3.751 (0.692)	
Range	3.333–5.000	2.333–4.667	1.000–3.500	1.000–5.000	
Critical thinking					< 0.001
Mean (SD)	4.436 (0.447)	3.508 (0.473)	2.519 (0.647)	3.713 (0.819)	
Range	3.000–5.000	1.750–5.000	1.000–4.000	1.000–5.000	
Initiative					< 0.001
Mean (SD)	4.254 (0.435)	3.382 (0.488)	2.511 (0.595)	3.583 (0.771)	
Range	2.800–5.000	1.600–4.800	1.000–3.600	1.000–5.000	
Teamwork					< 0.001
Mean (SD)	4.476 (0.447)	3.896 (0.479)	3.053 (0.709)	3.991 (0.693)	
Range	2.600–5.000	2.200–5.000	1.000–4.600	1.000–5.000	
Networking					< 0.001
Mean (SD)	4.480 (0.457)	3.795 (0.524)	2.968 (0.673)	3.932 (0.731)	
Range	2.250–5.000	2.250–5.000	1.000–4.750	1.000–5.000	

### Multinomial logistic regression analysis

Multinomial logistic regression analysis was performed to investigate how resilience, self-monitoring, and educational level relate to innovative abilities, which were built as a series of latent profiles. The referent group was profile 1 with strong innovative abilities in this sample. As evident in Table 5, both self-monitoring and resilience mean scores negatively predicted the likelihood of being a member of profile 2 and 3, relative to the reference category, supporting hypotheses 1 and 2. In other words, the increase in self-monitoring and resilience are associated with the memberships in profile 1 with the strong innovative abilities. Moreover, college students were more likely than university undergraduates to be members of the weak profile compared to the strong profile. However, it is not the case between moderate and strong profiles - where the educational level is insignificant.

**Table 5:** Predicting profile membership by self-monitoring, resilience, and educational level

	Profile 2 Moderate innovative abilities		Profile 3 Weak innovative abilities	
*Predictors*	*Coefficients (Odds Ratios)*	*std. Error*	*Coefficients (Odds Ratios)*	*std. Error*
Self-monitoring	-0.11 (0.90) ^***^	0.03	-0.13 (0.88) ^**^	0.04
Resilience	-0.51 (0.60) ^***^	0.06	-0.71 (0.49) ^***^	0.06
Education level [University − 1]	-0.3 (0.74)	0.14	-0.9 (0.41) ^**^	0.11

p < 0.05   ** p < 0.01   *** p < 0.001

## Discussion

This study extends the innovative work behavior concept through exploring the dimensions of innovation competence across college and university students in the form of unique profiles. Using the LPA, this study identified three distinct categories of students in terms of the composition of innovative abilities. Thus, the first contribution of this article is that it linked students with similar patterns of innovation competence, who differed from the composition observed in other groups based on the FINCODA typology, which reflects problem recognition, idea generation, promotion, and implementation abilities. Our findings offered three interpretable innovation competence profiles with strong (37.8%), moderate (47.5%) and weak (14.7%) innovative abilities. The strong sub-group characterizes participants with a comparatively high level of all five dimensions of the FINCONDA innovation competence model. On the other extreme, the weak profile typifies students who scored low on the innovative capabilities required for the innovation phases, particularly the critical thinking and initiative dimensions. Quite informatively, female students (90.4%) and those with college education predominate the weak sub-group. Thus, education and human resource practitioners, as well as education policy makers, should introduce various strategies when planning development and training interventions aimed at bolstering employees’ and students’ creativity and innovativeness.

Next, this study attempts to identify how personality, by way of resilience and self-monitoring, impacts the membership in innovation competence profiles. Drawing on the componential theory of creativity, we introduced a framework where individual resilience and self-monitoring influence the affiliation with a particular level of innovative abilities in the student population. This study confirmed the empowering role of personality characteristics of individuals (resilience and self-monitoring) as predictors of students’ membership into the innovation competence profiles.

Specifically, we find that individual resilience leads to either higher or lower levels of innovative abilities. In other words, highly resilient students are more likely to belong to the strong innovative abilities profile, and less likely to fall into the moderate and weak classes compared to those with a low level of resilience. This finding is consistent with other recent studies [19], [20], [49], reporting the positive role of resilience in innovative work behavior and employee’s creative abilities. The mindfulness-based interventions such as taking an interest in each experience with acceptance and openness [50] may be a successful strategy to improve the students’ resilience level [31]. Thus, promoting individual resilience through, for instance, the development of mindfulness, may help to increase student abilities to adopt innovative work behavior.

Self-monitoring also increases the odds of membership into the stronger innovation competence profiles. Put differently, students that are high-self monitors are predicted to be more likely to belong to the strong innovative group, and less likely to be a member of moderate and weak categories compared to the low self-monitor students. This finding supports that the willingness to involve in innovative behavior depends on individual differences in self-monitoring [13]. Additionally, this result resonates with the findings where high-self monitors are more receptive to improvement feedback from their superior which they in turn use to develop their creative capabilities [39]. University undergraduates and college students are social beings with very strong motives to gain acceptance and recognition. This strong need for image gains can drive them to increase their level of team co-operation and creativity [13] and, by extension, their membership of the strong innovative abilities class. In so doing, educational strategies should include both social support and delicate feedback from instructors, peers, or representatives of the leading industry to enhance student motivation in innovative activities.

Finally, while education is not a significant predictor of membership into the moderate innovative abilities profile, it does for the weak class. This implies that students with a higher level of education are more likely to belong to the strong innovation profile, and less likely to be in the weak profile compared to those with lower levels of education. Further, the weak innovative abilities sub-group accounted for 73.4% of college students. This implies that the former are more at a disadvantage relative to their university peers in terms of innovative abilities and might require intervention to close their deficit competence gap. Similarly, this sub-group accounted for 90.4% of females Hence, enrollment in resilience-building interventions should be prioritized for female and college students who have limited innovative abilities.

### Implications for theory and practice

This study has several implications for theory and practice. *As for theoretical contribution*, the current research extended the existing literature on individual innovative behavior by using LPA to investigate profiles of innovative abilities and discover their unique patterns in college and university students. Specifically, the study found three sub-groups (innovation competence profiles), differentiated by a strong, moderate, or weak level of innovative abilities. This result provides preliminary evidence of the group sensitivity of innovation competence configurations. Next, we documented that the individual cognitive ability to face and overcome adversity (resilience), as well as surface acting (self-monitoring), a social skill, are significant determinants of membership of these distinct profiles. Overall, the study contributes to the literature on innovative work behavior by identifying distinct innovation competence profiles and their antecedents.

*As for practical implications*, organizations can profile the unique innovative abilities of present and potential employees for further development, in line with the most recent trend focuses on assessing candidates’ abilities rather than prior experience and merits [51]. Specifically, employee profiling that includes their innovation competence category might be useful to Human Resource managers in their training interventions. For example, while planning for such interventions, they could collate data about the innovation competence level of their employees and then categorize them in any of the three profiles. After profiling, the intervention and its content should be tailored to meet the specific training needs of the employees as reviewed by their group competence profile.

The insight from the study might also improve the recruitment process because resilience has been identified as a beneficial characteristic of innovators [49], [52]. As such, Human Resource managers might also want to prioritize candidates with a high level of resilience, if the employers aim to increase their organization pool of innovators. Practically, the Brief Resilience Scale could be a handy tool here as a standard measure of resilience in recruiter’s selection materials.

However, given that the study establishes self-monitoring and individual resilience as positive antecedents of membership in innovation competence profiles, therefore we also recommend several ways to bolster them. These personal characteristics are closely related to the social-organizational work environment, and can be improved, among other things, via various social discourses (e.g., storytelling) [53]. Storytelling contributes to the development of individual resilience of storytellers and listeners [32]. As such, social discourses, such as narrative stories or examples, told by members of a strong innovation competence profile, about personal experiences in activities related to innovations and their hardiness can be an effective source of information for others. Interventions can take the form of collecting and distributing these discourses via class activities or social media. Doing this can help managers and instructors to strengthen the level of individual resilience and create a work environment with emphasis on innovations.

Furthermore on resilience building, employees involved in innovation activities leverage their social networks as external loci of support (asking for help, shared responsibility in problem solving) in line with King and others [52]. Emotional support received from such networks has been documented as a booster source of innovator resilience. In so doing, managers and instructors should stimulate social bonds between individuals with strong innovative abilities and the two remaining sub-groups. This can be achieved through interventions based on peer-to-peer learning (like introducing peer coaching or communities of practice).

### Limitations and future research

This study has several limitations. First, we measured only one out of the two dimensions of the self-monitoring concept; the individual ability to adapt their behavior to the situation. However, the motives to get ahead of others, which is the personal values dimension of the self-monitoring concept could equally be measured and investigated in further research studies. Second, self-reported questionnaires may not be free from biases as respondents may tend to over or underestimate their own abilities. Future investigation might consider using third-party raters, e.g., peers, instructors, co-workers, supervisors etc. Third, a relatively small sample size limits the scope of this study, but 638 number of participants is acceptable based on the previous research [5], [8]. Further, this sample shares a similar culture with the majority coming from the old Soviet Union. It will be interesting to know what effect cultural differences would have on the findings of this study. We call on future research to investigate the moderating role of culture using larger and wider samples of respondents from different emerging economies by using longitudinal design.

## Conclusion

The aim of this research is to investigate the innovation competence profiles of students from various educational levels and study the role of individual resilience and self-monitoring in predicting the memberships of these profiles. The latent profile analysis revealed that students can be classified into three profiles—strong, moderate, and weak—based on how innovative they are. Further, high levels of resilience and self-monitoring predict membership into the profile with strong innovative abilities. Hence, education practitioners might find it useful to first profile their students (incoming and current) by their innovative abilities as a way to enrich their curriculum design and development. For example, courses aimed at teaching innovation should contain content on how to build resilience and include social support and feedback. The college education curriculum should really emphasize learning outcomes like creativity, critical thinking teamwork, initiatives, and networking. This is particularly important because of the strategic role of college education in promoting entrepreneurship among the youths.

## Data Availability

The datasets used and analyzed during the current study are available from the corresponding author on reasonable request.

## List of Abbreviations

FINCODA: Framework for Innovation Competencies Development and Assessment
HSM: High Self-Monitors
LSM: Low Self-Monitors
RMSEA: Root-mean-square error of approximation
TLI: Tucker Lewis Index
CFI: Comparative fit index
SRMR: Standardized Root Mean Square Residual
LPA: *Latent Profile Analysis*
BIC: Bayesian information criterion
AIC: Akaike Information Criterion
CAIC: Consistent AIC
BLRT: Bootstrapped likelihood ratio test
MGCFA: Multigroup confirmatory factor analysis
